# Epigallocatechin-3-Gallate Attenuates Leukocyte Infiltration in 67-kDa Laminin Receptor-Dependent and -Independent Pathways in the Rat Frontoparietal Cortex following Status Epilepticus

**DOI:** 10.3390/antiox12040969

**Published:** 2023-04-20

**Authors:** Ji-Eun Kim, Duk-Shin Lee, Tae-Cheon Kang

**Affiliations:** Department of Anatomy and Neurobiology, Institute of Epilepsy Research, College of Medicine, Hallym University, Chuncheon 24252, Republic of Korea

**Keywords:** 67LR, CCR2, ERK1/2, MCP-1, MIP-2, monocyte, neutrophil, U0126

## Abstract

Status epilepticus (SE) evokes leukocyte infiltration in the frontoparietal cortex (FPC) without the blood-brain barrier disruption. Monocyte chemotactic protein-1 (MCP-1) and macrophage inflammatory protein-2 (MIP-2) regulate leukocyte recruitments into the brain parenchyma. Epigallocatechin-3-gallate (EGCG) is an antioxidant and a ligand for non-integrin 67-kDa laminin receptor (67LR). However, it is unknown whether EGCG and/or 67LR affect SE-induced leukocyte infiltrations in the FPC. In the present study, SE infiltrated myeloperoxidase (MPO)-positive neutrophils, as well as cluster of differentiation 68 (CD68)-positive monocytes in the FPC are investigated. Following SE, MCP-1 was upregulated in microglia, which was abrogated by EGCG treatment. The C–C motif chemokine receptor 2 (CCR2, MCP-1 receptor) and MIP-2 expressions were increased in astrocytes, which were attenuated by MCP-1 neutralization and EGCG treatment. SE reduced 67LR expression in astrocytes, but not endothelial cells. Under physiological conditions, 67LR neutralization did not lead to MCP-1 induction in microglia. However, it induced MIP-2 expression and extracellular signal-regulated kinase 1/2 (ERK1/2) phosphorylation in astrocytes and leukocyte infiltration in the FPC. Co-treatment of EGCG or U0126 (an ERK1/2 inhibitor) attenuated these events induced by 67LR neutralization. These findings indicate that the EGCG may ameliorate leukocyte infiltration in the FPC by inhibiting microglial MCP-1 induction independent of 67LR, as well as 67LR-ERK1/2-MIP-2 signaling pathway in astrocytes.

## 1. Introduction

The brain is in part isolated from the systemic immune system by the blood-brain barrier (BBB). Therefore, microglia generally act as the primary immune cells in the brain parenchyma. Under pathophysiological conditions, activated microglia lead to blood-derived leukocyte infiltration by releasing various cytokines and chemokines. Infiltrating leukocytes further exacerbate secondary local inflammation by generating reactive oxygen species, proteolytic enzymes and cytokines/chemokines [1,2,3,4,5].

BBB disruption is a crucial step in the pathogenesis of several neuroinflammatory diseases in the brain [6]. Indeed, status epilepticus (SE), prolonged and uncontrolled seizures, results in the infiltration of neutrophil and monocyte in the rat piriform cortex (PC), accompanied by severe vasogenic edema [7]. Unlike the PC, SE evokes leukocyte infiltration in the frontoparietal cortex (FPC) without BBB breakdown. In FPC, SE induces leukocyte infiltration through inductions of monocyte chemotactic protein-1 (MCP-1) in microglia and macrophage inflammatory protein-2 (MIP-2) in astrocytes, in an interleukin-1β (IL-1β)-independent manner [8,9,10]. Therefore, the FPC is a suitable region to investigate the underlying mechanisms of leukocyte infiltration unaffected by altered vascular permeability following SE.

Epigallocatechin-3-gallate (EGCG) is an antioxidant and an anti-inflammatory substance. EGCG attenuates immune cell infiltration in an experimental autoimmune encephalitis model, by inhibiting the p38 mitogen-activated protein kinase (p38 MAPK) and nuclear factor-κB (NF-κB) activity [11,12,13,14,15]. Furthermore, EGCG is a ligand of non-integrin 67 kDa laminin receptor (67LR) [16,17]. This receptr is composed of a 37 kDa precursor and expresses in some neurons, astrocyte and endothelial cells [10,16,17,18,19,20]. It modulates extracellular signal-regulated kinase 1/2 (ERK1/2), p38 MAPK and NF-κB signaling pathways [21,22,23]. Considering the requirement of p38 MAPK and NF-κB for SE-induced monocyte infiltration in the FPC [9,10], it is likely that EGCG and/or 67LR may affect SE-induced leukocyte infiltration, which is largely unknown. Therefore, the present study was conducted to investigate whether EGCG influences leukocyte infiltration through 67LR-mediated pathways.

Here, we demonstrate that SE infiltrated myeloperoxidase (MPO)-positive neutrophil, as well as cluster of differentiation 68 (CD68)-positive monocytes in the FPC. Following SE, microglia showed MCP-1 upregulation, which was abrogated by EGCG treatment. Astrocytes exhibited increased C–C motif chemokine receptor 2 (CCR2, MCP-1 receptor) and MIP-2 expressions, concomitant with reduced 67LR expression. These astroglial responses were attenuated by MCP-1 neutralization and EGCG treatment. Under physiological condition, 67LR neutralization increased ERK1/2 phosphorylation and MIP-2 expression in astrocytes, and resulted in MCP-1-independent leukocyte infiltration. These 67LR neutralization-induced events were abolished by EGCG and U0126 (an ERK1/2 inhibitor). Therefore, these findings indicate that MCP-1 may regulate CCR2-67LR-ERK1/2-MIP-2 signaling pathway in astrocytes during leukocyte infiltration, and that EGCG may ameliorate neuroinflammation in 67LR-dependent (in astrocyte) and -independent (in microglia) manners.

## 2. Materials and Methods

### 2.1. Experimental Animals and Chemicals

Male Sprague-Dawley rats (7 weeks old, 200–220 g), purchased from Daehan biolink (Umseong, Chungcheongbuk-do, South Korea), were used in all experiments. Animals were housed under a 12 h dark/light cycle with free access to food and water. All experimental procedures were approved by the Institutional Animal Care and Use Committee of Hallym University (Hallym 2021-3, approval date: 17 May 2021). All reagents were obtained from Sigma-Aldrich (St. Louis, MO, USA), except as noted.

### 2.2. Surgical Procedures and SE Induction

Rats were implanted with a brain infusion kit 1 (Alzet, Cupertino, CA, USA) into the right lateral ventricle (coordinates: 1 mm posterior; 1.5 mm lateral; 3.5 mm depth) under Isoflurane anesthesia (3% induction, 1.5–2% for surgery, and 1.5% maintenance in a 65:35 mixture of N_2_O:O_2_). Thereafter, an Alzet 1007D osmotic pump (Alzet, Cupertino, CA, USA) containing (1) vehicle, (2) EGCG (50 μM), (3) control anti-mouse IgG (Abcam, #ab37355, Cambridge, UK, 50 μg/mL) or (4) anti-MCP-1 IgG (Abcam, #ab25124, Cambridge, UK, 50 μg/mL) was connected to an infusion kit and infused for over 7 days. The infusion needle was secured to the exposed skull with a dental acrylic. The correct location of the infusion needle into the ventricle was confirmed during brain sections. In pilot and previous studies [17], each treatment did not evoke neurological adverse effects and alter the seizure susceptibility and its severity in response to pilocarpine. Two days after surgery, animals were treated with LiCl (127 mg/kg, i.p.). The next day, 20 min before pilocarpine administration, atropine methylbromide (5 mg/kg i.p.) was injected. Thereafter, animals were given pilocarpine (30 mg/kg, i.p.). Two hours after SE onset, diazepam (Valium; Hoffmann-la Roche, Neuilly-sur-Seine, France; 10 mg/kg, i.p.) was injected to cease seizure activity and repeated, as needed. Control animals received saline substituted for pilocarpine.

### 2.3. 67LR Neutralization

Animals were implanted with a brain infusion kit (Alzet, Cupertino, CA, USA) into the right lateral ventricle by the same method described above. Thereafter, an Alzet 1003D osmotic pump (Alzet, Cupertino, CA, USA) containing (1) control anti-rabbit IgG (Abcam, #ab37415, Cambridge, UK, 50 μg/mL) + vehicle, (2) anti-67LR IgG (Abcam, #ab133645, UK, 50 μg/mL) + vehicle, (3) anti-67LR IgG (50 μg/mL) + EGCG (50 μM) or (4) anti-67LR IgG (50 μg/mL) + U0126 (25 μM) was connected to an infusion kit and infused for over 3 days [19,20,24].

### 2.4. Tissue Preparation and Immunohistochemistry

Since the neutrophil and monocyte infiltrations peaked in the PFC at 2–3 days and 3–4 days after SE, respectively [8,9,10], we chose 3 days after SE as the ideal timepoint to evaluate the effect of EGCG on leukocyte infiltration. Three days after SE or 67LR infusion, animals were administered urethane anesthesia (1.5 g/kg, i.p.) and perfused with normal saline followed by 4% paraformaldehyde in 0.1 M phosphate buffer (PB, pH 7.4). The brains were collected in the same fixative overnight and 30 μm thick coronal sections were made using a cryostat. Sections were blocked with 3% bovine serum albumin and subsequently incubated with a cocktail solution containing isolectin B4 (IB4) or primary antibodies (Table 1) overnight at room temperature. Thereafter, sections were reacted with Brilliant Violet-, Cy2- or Cy3-conjugated secondary antibodies (for anti-sera) or streptavidin (for IB4). A negative control test was performed with pre-immune serum in place of the primary antibody. Experimental procedures in this study were carried out under the same conditions and in parallel. The random-selected areas (1 × 10^5^ μm^2^), approximately −3.0–3.6 mm from the bregma, were selected based on the rat brain in stereotaxic coordinates [25]. Thereafter, the number of infiltrating neutrophils and monocytes was counted, and SMI-71 fluorescent intensity was measured using AxioVision Rel. 4.8 and the ImageJ software (*n* = 7 rats in each group). The fluorescent intensity of MCP-1, MIP-2, 67LR or p-ERK1/2 was also measured in randomly selected 5–6 cells from each animal. Briefly, images were captured (gain value = 1) and digitally separated into green, red or blue panels. Each image was converted to black and white, and the background staining was subtracted automatically. Thereafter, each signal was normalized by setting the threshold level and represented as the number of 256 grayscale. Manipulation of the microscope was restricted to automatic exposure time and threshold adjustments.

### 2.5. Western Blot and Quatitative Real-Time PCR (qRT-PCR)

Three days after SE and 67LR IgG infusion, animals were decapitated and the FPC was rapidly obtained. Western blot was performed by the standard methods. Briefly, the proteins were separated by electrophoresis and transferred to nitrocellulose membranes. After the blocking, membranes were incubated with primary antibody (Table 1). After further reaction with peroxidase-conjugated secondary antibody followed by ECL solution, immunobands were detected and quantified with the ImageQuant LAS4000 system (GE Healthcare Korea, Seoul, South Korea). The density of the immunobands was calibrated with with β-actin. The ratio of phospho-protein to total protein was also measured. For qRT-PCR, brain tissues were homogenized, and the total RNA was extracted using Trizol Reagents (Thermo Fisher Scientific Korea, Seoul, South Korea). Total RNA was reverse-transcribed into first-strand cDNA using the PrimerScript 1st strand cDNA synthesis kit (Takara, Shiga, Japan). Quantification of mRNA expression was performed in triplicate using a SYBR Green SuperMix (Bioneer, Taejon, South Korea) and with the MyiQ Single-Color Real-Time PCR Detection System (Bioneer, Taejon, South Korea). Primer sequences were 5′-GTGCTGACCCCAATAAGGAA-3′ (forward primer for rat MCP-1) and 5′-TGAGGTGGTTGTGGAAAAGA-3′ (reverse primer for rat MCP-1); 5′-TGAAGTTTGTCTCAACCCTGAAGCC-3′ (forward primer for rat MIP-2) and 5′-AGGTCAGTTAGCCTTGCCTTTGTTC-3′ (reverse primer for rat MIP-2); and 5′-TGGAGTCTACTGGCGTCTT-3′ (forward primer for rat GAPDH) and 5′-TGTCATATTTCTCGTGGTTCA-3′ (reverse primer for rat GAPDH). All primers were purchased from Bioneer (Taejon, South Korea). After initial denaturation at 95 °C for 10 min, 50 cycles of primer annealing and elongation were conducted at 55 °C for 45 s, followed by denaturation at 95 °C for 1 s. qRT-PCR data for MCP-1 and MIP-2 were normalized to GAPDH determined from the same experiment.

### 2.6. Data Analysis

Data were analyzed using a Mann–Whitney test or Kruskal–Wallis test, followed by Dunn–Bonferroni *post hoc* comparison. A *p*-value of less than 0.05 was considered significant.

## 3. Results

### 3.1. EGCG Attenuates SE-Induced Leukocyte Infiltration in the FPC

**Figure 1:**
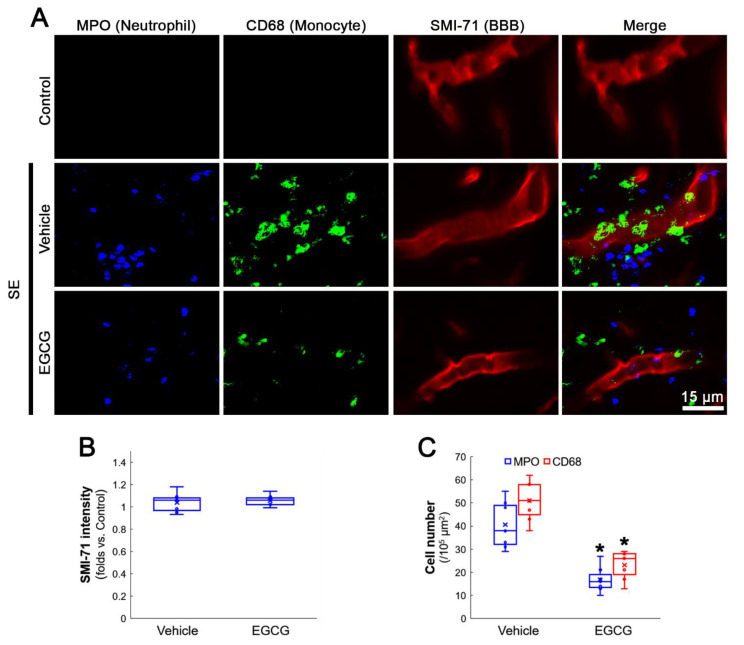
Effects of EGCG on leukocyte infiltration in the FPC following SE. SE leads to leukocyte infiltration without altering the BBB integrity. As compared to the vehicle, EGCG attenuates neutrophil and monocyte infiltration induced by SE. (**A**) Representative photos of neutrophil (MPO), monocytes (CD68) and the BBB integrity (SMI-71) in the FPC. (**B**) Quantification of SMI-71 intensity. (**C**) Quantification of the number of infiltrating leukocytes. * *p* < 0.05 vs. vehicle (*n* = 7 rats, respectively).

First, we investigated whether EGCG treatment affects leukocyte infiltration in the FPC following SE. SE did not alter the BBB (SMI-71) integrity in the FPC (*Z* = 0.513, *p* = 0.608, *n* = 7 rats, respectively, Mann–Whitney test; Figure 1A,B). In control (non-SE) animals, MPO-positive neutrophils were undetectable in the parenchyma of the FPC (Figure 1A). Following SE, the number of MPO-positive neutrophils was ~41 cells/10^5^ μm^2^ in the FPC of vehicle-treated rats. EGCG attenuated neutrophil infiltration to ~17 cells/10^5^ μm^2^ in this region (*Z* = 3.130, *p* = 0.002, *n* = 7 rats, respectively, Mann–Whitney test; Figure 1A,C). Similar to MPO-positive neutrophils, CD68-positive monocytes were rarely observed in the FPC of control animals (Figure 1A). Following SE, round-, spheroid- and ramified-shaped CD68-positive monocytes were detected in the FPC (Figure 1A). The number of CD68-positive monocytes was ~51 cells/10^5^ μm^2^ in the FPC of vehicle-treated rats. EGCG attenuated monocyte infiltration to ~23 cells/10^5^ μm^2^ in this region (*Z* = 3.137, *p* = 0.002, *n* = 7 rats, respectively, Mann–Whitney test; Figure 1A,C). Furthermore, the shape of the infiltrating monocytes was round or spheroid, rather than ramified (Figure 1A). These findings indicate that EGCG may ameliorate leukocyte infiltration in the FPC following SE. Considering that blood-derived monocytes replace the resident microglia [8,26,27], our findings also suggest that EGCG may inhibit monocyte transformation to microglia.

### 3.2. EGCG Ameliorates MCP-1 and MIP-2 Expression in the FPC following SE

MCP-1 is a chemokine to recruit monocytes into the brain parenchyma [28,29,30,31]. Furthermore, MCP-1/CCR2 signaling regulates MIP-2 expression that is required for neutrophil infiltration [32,33]. Following SE, MCP-1 is induced in microglia, while MIP-2 is detected in astrocytes and neurons in the FPC [8]. Therefore, we validated the effect of EGCG on SE-induced MCP-1, CCR2 and MIP-2 inductions in the FPC following SE.

**Figure 2:**
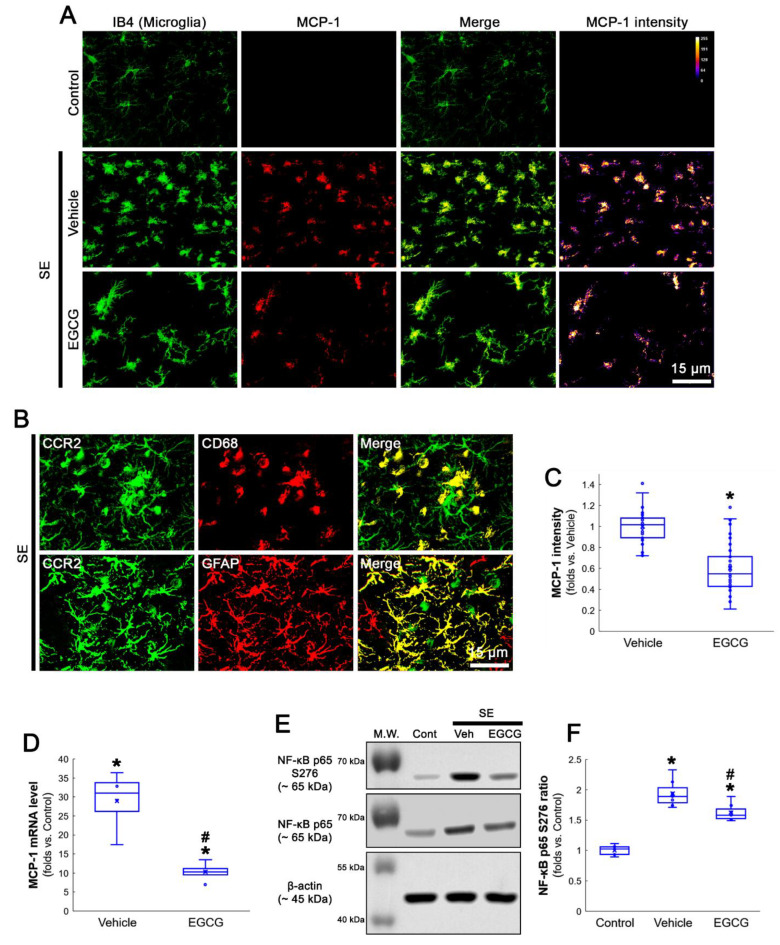
SE-induced microglial activation of MCP-1 induction and cellular localization of CCR2 (MCP-1 receptor). SE results in MCP-1 upregulation in activated microglia, which are ameliorated by EGCG. In addition, CCR2 expression is detected in infiltrating monocytes and astrocytes following SE. (**A**) Representative photos of microglia (IB4), MCP-1 expression and MCP-1 intensity in the FPC. (**B**) Representative photos of cellular localization of CCR2 in monocyte and astrocytes. (**C**) Quantification of the MCP-1 intensity in microglia. * *p* < 0.05 vs. vehicle (*n* = 40 cells in 7 rats, respectively). (**D**) Quantification of the MCP-1 mRNA in the hippocampus. *^,#^ *p* < 0.05 vs. control and vehicle (*n* = 4 rats, respectively). (**E**,**F**) Representative Western blot data for NF-κB p65 S276 phosphorylation and quantification of the NF-κB p65 S276 ratio in the hippocampus. *^,#^ *p* < 0.05 vs. control animal and vehicle, respectively (*n* = 7 rats, respectively).

In control animals, MCP-1 expression was rarely detected in the FPC (Figure 2A). Following SE, MCP-1 expression was observed in most hypertrophic and amoeboid isolectin B4 (IB4)-positive microglia (an indicative of activated microglia, Figure 2A). CCR2 expression was mainly detected in CD68-positive monocytes, as well as astrocytes (Figure 2B). In EGCG-treated animals, microglia showed hyper-ramified processes that were covered by thorny spines, indicating the inhibition of microglial transformation to hypertrophic and amoeboid shapes (Figure 2A). EGCG also reduced MCP-1 expression in microglia (*Z* = 6.228, *p* < 0.001, *n* = 40 cells in 7 rats, respectively, Mann–Whitney test; Figure 2A,C). Compatible with immunohistochemistry, the qRT-PCR date revealed that EGCG effectively diminished MCP-1 mRNA level (*Z* = 2.309, *p* = 0.021, *n* = 4 rats, respectively, Mann–Whitney test; Figure 2D). Since serine (S) 276 phosphorylation of NF-κB p65 subunit is required for microglial activation and MCP-1 induction [9,10], the effects of EGCG on NF-κB p65 S276 phosphorylation were also investigated. SE significantly increased the NF-κB p65 S276 ratio in the hippocampus following SE, which was attenuated by EGCG (*χ^2^*_(2)_ = 16.207, *p* < 0.001, Kruskal–Wallis test with Dunn–Bonferroni *post hoc* test, *n* = 7 rats, respectively; Figure 2E,F and Supplementary Figure S1). These findings indicate that EGCG may mitigate MCP-1 expression by inhibiting the NF-κB-mediated pathway following SE.

**Figure 3:**
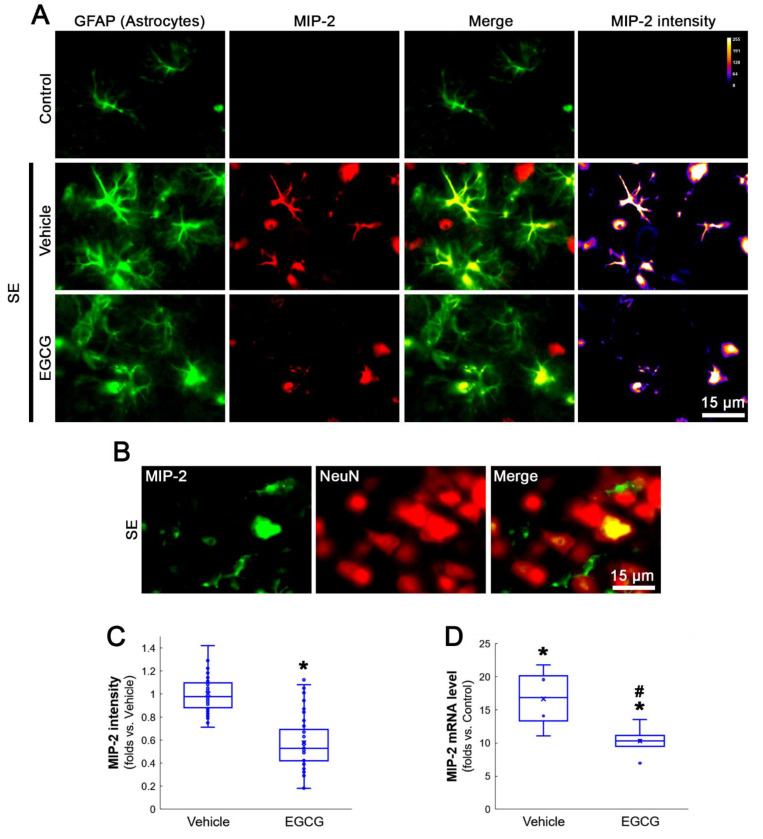
SE-induced MIP-2 induction and cellular localization of MIP-2. SE induces MIP-2 expression in reactive astrocytes and a few neurons. EGCG attenuates astroglial MIP-2 upregulation induced by SE. (**A**) Representative photos of astrocytes (GFAP), MIP-2 expression and MIP-2 intensity in the FPC. (**B**) Representative photos of CCR2 expression in a few neurons. (**C**) Quantification of the MIP-2 intensity in astrocytes. * *p* < 0.05 vs. vehicle (*n* = 40 cells in 7 rats, respectively). (**D**) Quantification of the MIP-2 mRNA in the hippocampus. *^,#^ *p* < 0.05 vs. control animal and vehicle, respectively (*n* = 4 rats, respectively).

On the other hand, MIP-2 expressions were observed in most astrocytes and a few neurons following SE, although its MIP-2 expression was undetected in the FPC of control rats (Figure 3A,B). EGCG abolished SE-induced MIP-2 induction in these cell populations (*Z* = 6.237, *p* < 0.001, *n* = 40 cells in 7 rats, respectively, Mann–Whitney test; Figure 3A,C). EGCG also inhibited the SE-induced MIP-2 mRNA upregulation in the hippocampus (*Z* = 2.309, *p* = 0.021, *n* = 4 rats, respectively, Mann–Whitney test; Figure 3D). Taken together, our findings indicate that EGCG may diminish NF-κB-mediated MCP-1 induction in microglia following SE, which abolished CCR2-mediated MIP-2 expression in astrocytes.

### 3.3. SE Reduces 67LR Expression in Astrocytes, but Not in Endothelial Cells, in the FPC

**Figure 4:**
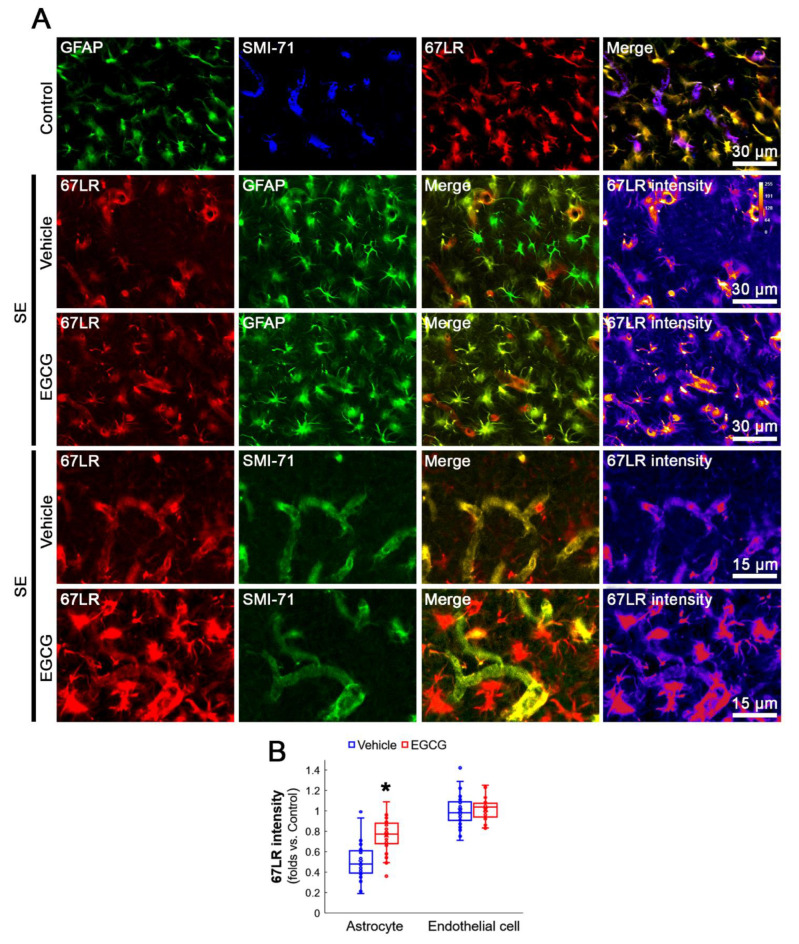
Effects of EGCG on 67LR expression in the FPC following SE. The 67LR expression is observed in astrocyte and endothelial cells in control animals. Following SE, 67LR expression is reduced in astrocytes, but not in endothelial cells, which is attenuated by EGCG. (**A**) Representative photos of GFAP, SMI-71 and 67LR in the FPC. (**B**) Quantification of 67LR intensity in astrocyte and endothelial cells. * *p* < 0.05 vs. vehicle (*n* = 40 cells in 7 rats, respectively).

Since EGCG is a 67LR ligand [16,17], we investigated whether SE alters 67LR expression in the FPC. Compatible with previous studies [17,19,20,24], 67LR expression was observed in astrocytes and endothelial cells in the FPC (Figure 4A). Following SE, 67LR expression was diminished in astrocytes, which was ameliorated by EGCG (*Z* = 5.771, *p* < 0.001, *n* = 40 cells in 7 rats, respectively, Mann–Whitney test; Figure 4A,B). However, SE did not change 67LR expression in endothelial cells, which was unaffected by EGCG (*Z* = 0.583, *p* = 0.56, *n* = 40 cells in 7 rats, respectively, Mann–Whitney test; Figure 4A,B). Therefore, it is likely that EGCG may attenuate SE-induced 67LR downregulation in astrocytes.

### 3.4. MCP-1 Neutralization Abolishes MIP-2 Expression, Leukocyte Infiltration and 67LR Downregulation Induced by SE

**Figure 5:**
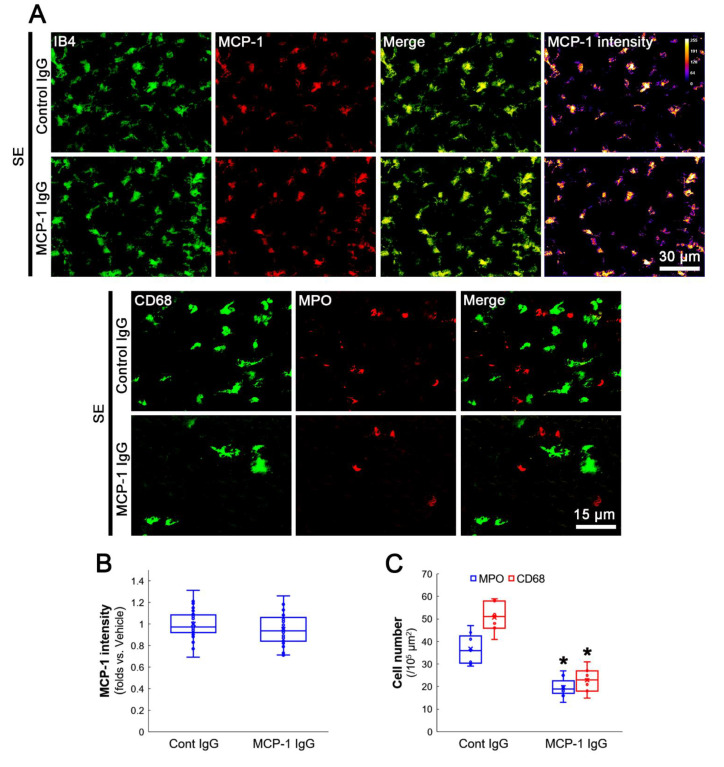
Effects of MCP-1 neutralization on MCP-1 induction and leukocyte infiltration in the FPC following SE. As compared to control IgG, MCP-1 neutralization attenuates leukocyte infiltration induced by SE. (**A**) Representative photos of MCP-1 expression and leukocyte infiltration in the FPC following SE. (**B**) Quantification of the MCP-1 intensity in microglia (*n* = 40 cells in 7 rats, respectively). (**C**) Quantification of the number of infiltrating leukocytes. * *p* < 0.05 vs. control IgG (*n* = 7 rats, respectively).

**Figure 6:**
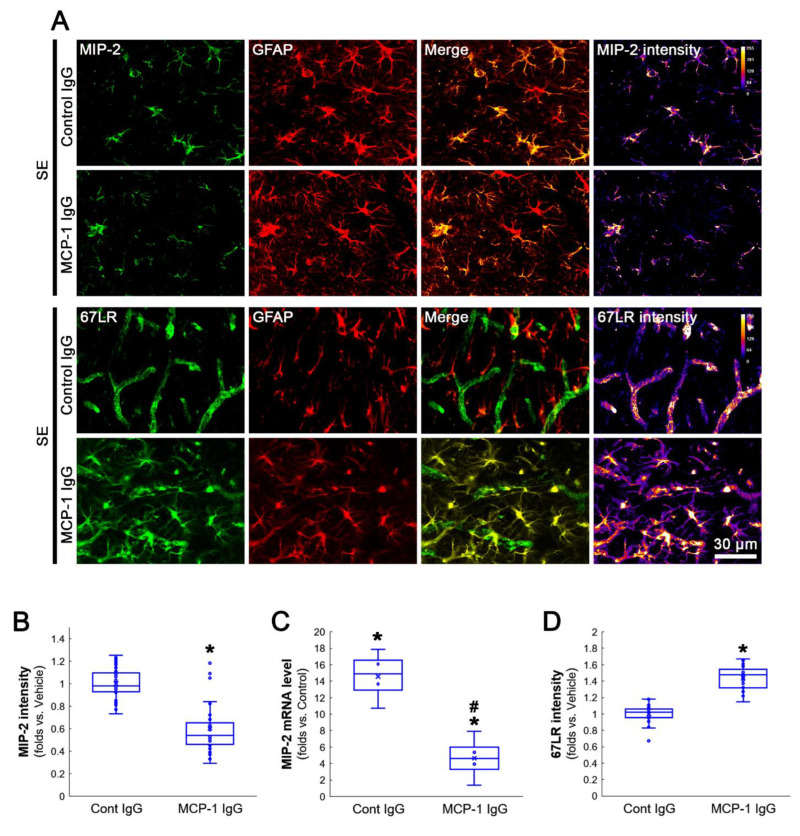
Effects of MCP-1 neutralization on MIP-2 and 67LR expressions in the FPC following SE. As compared to control IgG, MCP-1 neutralization ameliorates MIP-2 induction and increases 67LR expression in astrocytes. (**A**) Representative photos of MIP-2 and 67LR expressions in the FPC following SE. (**B**) Quantification of the MIP-2 intensity in astrocytes (*n* = 40 cells in 7 rats, respectively). * *p* < 0.05 vs. control IgG (*n* = 7 rats, respectively). (**C**) Quantification of the MIP-2 mRNA in the hippocampus. *^,#^ *p* < 0.05 vs. control animal and vehicle, respectively (*n* = 4 rats, respectively). (**D**) Quantification of 67LR intensity in astrocytes. * *p* < 0.05 vs. control IgG (*n* = 40 cells in 7 rats, respectively).

Next, we applied MCP-1 neutralization to confirm the role of MCP-1 in the regulation of MIP-2 and 67LR following SE. MCP-1 neutralization did not affect microglial activation and MCP-1 expression in the FPC following SE (*Z* = 1.613, *p* = 0.107, *n* = 40 cells in 7 rats, respectively, Mann–Whitney test; Figure 5A,B). However, MCP-1 neutralization effectively ameliorated SE-induced infiltration of neutrophils (*Z* = 3.13, *p* = 0.002, *n* = 7 rats, respectively, Mann–Whitney test; Figure 5A,C) and monocytes (*Z* = 3.13, *p* = 0.002, *n* = 7 rats, respectively, Mann–Whitney test; Figure 5A,C). MCP-1 neutralization abrogated MIP-2 induction in astrocytes (*Z* = 6.372, *p* < 0.001, *n* = 40 cells in 7 rats, respectively, Mann–Whitney test; Figure 6A,B) and MIP-2 mRNA expression in the hippocampus (*Z* = 2.309, *p* = 0.021, *n* = 4 rats, respectively, Mann–Whitney test; Figure 6C). In addition, MCP-1 neutralization restored 67LR in astrocytes (*Z* = 7.653, *p* < 0.001, *n* = 40 cells in 7 rats, respectively, Mann–Whitney test; Figure 6A,D). These findings indicate that MCP-1 released from microglia may evoke 67LR downregulation and MIP-2 induction in astrocytes.

### 3.5. Neutralization of 67LR Leads to ERK1/2-MIP-2-Mediated Leukocyte Infiltration in the FPC under Physiological Conditions

**Figure 7:**
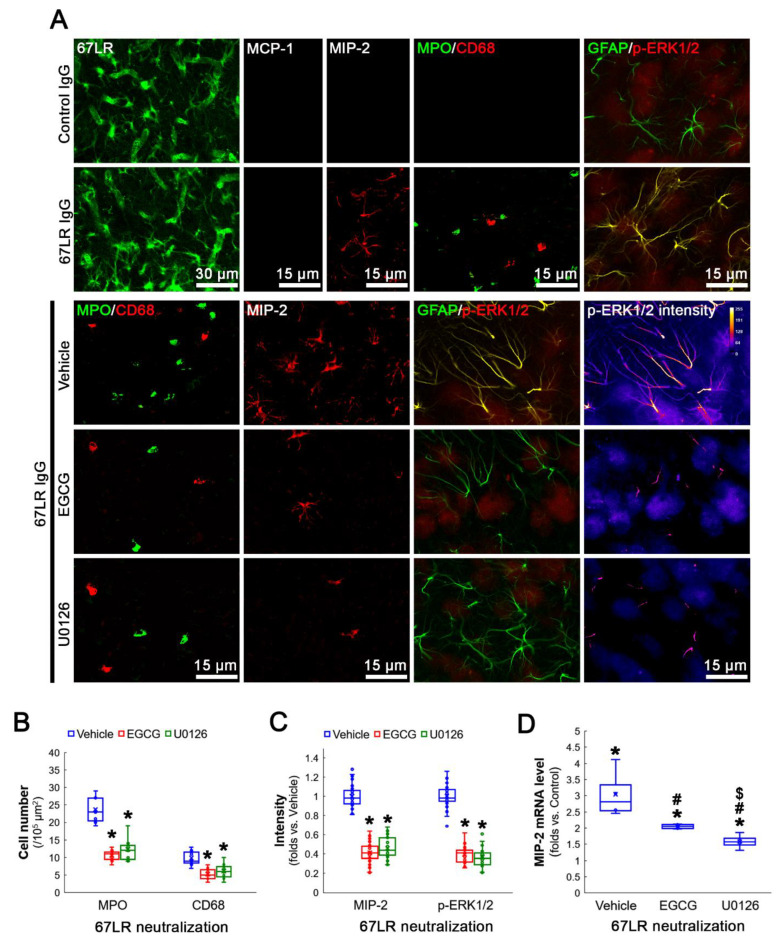
Effects of 67LR neutralization and co-treatment of EGCG or U0126 on leukocyte infiltration, MIP-2 expression and p-ERK1/2 level in the FPC under physiological condition. Although 67LR neutralization does not alter 67LR expression, it induces MIP-2 induction, leukocyte infiltration and p-ERK1/2 upregulation in the FPC without MCP-1 induction, which are ameliorates by co-treatment of EGCG or U0126. (**A**) Representative photos of 67LR, MCP-1, MIP-2 and p-ERK1/2 expressions in the FPC induced by 67LR neutralization. (**B**) Quantification of the number of infiltrating leukocytes. * *p* < 0.05 vs. vehicle co-treatment (*n* = 7 rats, respectively). (**C**) Quantification of MIP-2 and p-ERK1/2 levels in astrocytes. *^,#^*p* < 0.05 vs. co-treatment (*n* = 40 cells in 7 rats, respectively). (**D**) Quantification of the MIP-2 mRNA in the hippocampus. *^,#,$^ *p* < 0.05 vs. control, vehicle and EGCG, respectively (*n* = 4 rats, respectively).

To directly validate the role of astroglial 67LR downregulation in leukocyte infiltration, we also applied 67LR neutralization in control animals. Consistent with previous studies [19,20,24], 67LR neutralization did not change 67LR expression in astrocytes and endothelial cells (Figure 7A). However, 67LR neutralization evoked MIP-2 induction and leukocyte infiltration in the FPC without MCP-1 induction (Figure 7A). EGCG co-treatment attenuated the infiltration of neutrophils (*χ^2^*_(2)_ = 13.57, *p* = 0.001, Kruskal–Wallis test; *p* < 0.001, Dunn–Bonferroni *post hoc* test, *n* = 7 rats, respectively; Figure 7A,B) and monocytes (*χ^2^*_(2)_ = 9.822, *p* = 0.007, Kruskal–Wallis test; *p* = 0.003, Dunn–Bonferroni *post hoc* test, *n* = 7 rats, respectively; Figure 7A,B). EGCG co-treatment also mitigated astroglial MIP-2 induction induced by 67LR neutralization (*χ^2^*_(2)_ = 80.828, *p* < 0.001, Kruskal–Wallis test; *p* < 0.003, Dunn–Bonferroni *post hoc* test, *n* = 40 cells in 7 rats, respectively; Figure 7A,C). These findings indicate that 67LR downregulation/inhibition may be involved in leukocyte infiltration by inducing MIP-2 production in astrocytes, which would be ameliorated by EGCG co-treatment.

ERK1/2 is one of the upstream regulators of MIP-2 expression [34,35]. Neutralization by 67LR increases ERK1/2 activity in astrocytes [20,24]. In addition, EGCG inhibits MIP-2 production and ERK1/2 activation induced by lipopolysaccharide (LPS) treatment [36]. Therefore, it is likely that 67LR neutralization may result in astroglial MIP-2 induction mediated by ERK1/2 activation. To confirm this, we co-applied U0126 with 67LR antibody to inhibit ERK1/2 activity. Compatible with previous studies [20,24], 67LR neutralization enhanced ERK1/2 phosphorylation, indicating the increased kinase activity. U0126 co-treatment effective attenuated the upregulated phospho(p)-ERK1/2 level in astrocytes induced by 67LR neutralization (*χ^2^*_(2)_ = 80.896, *p* < 0.001, Kruskal–Wallis test; *p* < 0.001, Dunn–Bonferroni *post hoc* test, *n* = 40 cells in 7 rats, respectively; Figure 7A,C). U0126 co-treatment also ameliorated infiltration of neutrophils (*χ^2^*_(2)_ = 13.57, *p* = 0.001, Kruskal-Wallis test; *p* < 0.001, Dunn-Bonferroni *post hoc* test, *n* = 7 rats, respectively; Figure 7A,B) and monocytes (*χ^2^*_(2)_ = 9.822, *p* = 0.007, Kruskal–Wallis test; *p* = 0.015, Dunn–Bonferroni *post hoc* test, *n* = 7 rats, respectively; Figure 7A,B). In addition, U0126 co-treatment attenuated astroglial MIP-2 upregulation induced by 67LR neutralization (*χ^2^*_(2)_ = 80.828, *p* < 0.001, Kruskal–Wallis test; *p* < 0.001, Dunn–Bonferroni *post hoc* test, *n* = 40 cells in 7 rats, respectively; Figure 7A,C). EGCG co-treatment also diminished the p-ERK1/2 level (*p* < 0.001 vs. vehicle, *p* = 0.097 vs. U0126, Dunn–Bonferroni *post hoc* test, *n* = 40 cells in 7 rats, respectively; Figure 7A,C). qRT-PCR data revealed that co-treatment of EGCG or U0126 ameliorated MIP-2 mRNA upregulation induced by 67LR neutralization (*χ^2^*_(2)_ = 9.846, *p* = 0.007, Kruskal–Wallis test with Dunn–Bonferroni *post hoc* test, *n* = 4 rats, respectively; Figure 7D). These findings indicate that EGCG may suppress leukocyte infiltration through 67LR-ERK1/2-MIP-2 signaling pathway, independent of MCP-1.

## 4. Discussion

Infiltrating leukocytes develop detrimental inflammatory responses by releasing pro-inflammatory cytokines [37,38]. They also produce chemoattractants for the subsequent monocyte chemotaxis, such as human cationic antimicrobial protein (hCAP18, also known as LL-37) and cathepsin G [39,40]. Therefore, inhibition of leukocyte infiltration prevents or reduces the secondary brain damage induced by neuroinflammation [37].

MCP-1 and its receptor CCR2 is the first characterized chemokine system in humans [41]. MCP-1 activates monocyte recruitment by itself, and also promotes neutrophil infiltration [30,31,42,43]. In the brain, microglia rapidly induce MCP-1 expression in response to harmful stresses via NF-κB- and p38 MAPK signaling pathways that are also involved in microglial activation (transformation) [9,10,13,14,44]. Indeed, NF-κB p65 protein level increases in the brain following acute seizures [45,46,47]. Furthermore, p65 S276 phosphorylation of NF-κB p65 subunit is required for microglial activation and MCP-1 induction following SE, which plays an important role in leukocyte infiltration in the FPC [9,10]. Consistent with these reports, the present data showed that SE elevated total p65 protein and its S276 phosphorylation levels. Since SE enhanced p65 S276 phosphorylation more than the total p65 protein upregulation, the p65 phosphorylation ratio was increased as compared to the control animals. In addition, EGCG attenuated SE-induced leukocyte infiltration in the FPC by diminishing the total p65 protein, its S276 phosphorylation and MCP-1 expression in microglia. EGCG also inhibited the transformation of resident microglia and infiltrating monocytes in the brain parenchyma. Considering that EGCG abrogates p38 MAPK and NF-κB signaling pathways [11,12], the present data indicate that EGCG may inhibit microglial activation, and in turn, ameliorate MCP-1 transcription following SE.

The roles of 67LR in EGCG-mediated MCP-1 regulation in peripheral macrophages in response to LPS are controversial: EGCG induces MCP-1 expression through p38 MAPK-c-Jun NH2-terminal kinase (JNK) signaling axis following LPS treatment, which is abrogated by 67LR neutralization [48]. In contrast, EGCG suppresses MCP-1 expression in response to LPS via the ERK1/2, p38 MAPK, JNK and NF-κB pathways, which is abolished by 67LR neutralization [12]. In the present study, 67LR expression was mainly observed in astrocytes and endothelial cells, but not in microglia and neurons, in the intact brain. SE reduced 67LR expression in astrocytes. Furthermore, 67LR neutralization led to leukocyte infiltration without microglial MCP-1 induction under physiological conditions. Of note, EGCG diminished leukocyte infiltration following SE and 67LR neutralization. Therefore, our findings suggest that in the brain, EGCG may inhibit MCP-1 induction through NF-κB-dependent and 67LR-independent pathways in microglia following SE, unlike fully differentiated peripheral macrophages in response to LPS.

As aforementioned, MCP-1 also recruits neutrophils in MIP-2-dependent and -independent manners [30,42,43]. In a previous study [48], EGCG did not directly induce leukocyte migration, but induced MIP-2 in peripheral macrophages mediated by 67LR following LPS treatment. In contrast to this report, the present data demonstrate that SE resulted in astroglial MIP-2 induction with 67LR downregulation, which was mitigated by EGCG treatment and MCP-1 neutralization. These findings indicate that MCP-1 released from activated microglia may cause 67LR downregulation and the subsequent MIP-2 induction in astrocytes following SE. The present study also reveals that 67LR neutralization led to astroglial MIP-2 induction and leukocyte infiltration without microglial MCP-1 induction under physiological conditions, which was attenuated by EGCG co-treatment. These findings suggest that 67LR may play an inhibitory role in MIP-2-mediated leukocyte infiltration in the FPC.

The activation of MCP-1/CCR2 system enhances ERK1/2 activity in astrocytes that increases MIP-2 expression [34,35,49,50]. Interestingly, 67LR neutralization increases ERK1/2 activity in astrocytes [20,24]. In addition, EGCG inhibits MIP-2 production and ERK1/2 activation induced by the LPS [36]. Compatible with these previous studies, the present data show that 67LR neutralization increased ERK1/2 phosphorylation, which was abrogated by EGCG co-treatment. Furthermore, U0126 co-treatment ameliorated astroglial MIP-2 induction and leukocyte infiltration induced by 67LR neutralization. Thus, our findings suggest that 67LR downregulation/dysfunction may result in leukocyte infiltration via the ERK1/2-MIP-2 signaling pathway in astrocytes.

Exogenous soluble laminin and EGCG inhibit ERK1/2 phosphorylation mediated by 67LR [23,51]. Ku et al. [52,53] reported that 67LR neutralization abrogates the inhibitory effects of EGCG on ERK1/2 phosphorylation. Thus, they speculated that 67LR neutralization might prevent the EGCG-67LR interaction through sterical hindrance [52,53]. However, the present data demonstrate that 67LR neutralization increased ERK1/2 phosphorylation, which was attenuated by EGCG or U0126 co-treatment. Furthermore, both EGCG and U0126 attenuated leukocyte infiltration and ERK1/2 and MIP-2 upregulation induced by 67LR neutralization. In the present study, we used the antibody recognizing the amino acid 250–350 regions on 67LR for neutralization. Since amino acid 272–280 regions on 67LR are the inhibition site of its functions by neutralization [54] and 161–170 regions are the EGCG binding site, respectively [55], it is likely that 67LR neutralization may not affect EGCG–67LR binding in the present study. Adversely, EGCG and 67LR antiserum could competitively bind to 67LR in the present study.

In previous studies [9,10], we have reported that roscovitine, a cyclin-dependent kinase 5 (cdk5) inhibitor, attenuates SE-induced leukocyte infiltration in the FPC by inhibiting p38 MAPK [9]. Furthermore, 2-cyano-3,12-dioxo-oleana-1,9(11)-dien-28-oic acid methyl ester (CDDO-Me), a synthetic triterpenoid, also inhibits monocyte infiltration in this region by abrogating NF-κB- and p38 MAPK-mediated signaling pathways following SE [10]. Similar to CDDO-Me, EGCG attenuates immune cell infiltration by inhibiting p38 MAPK and NF-κB activity in an experimental autoimmune encephalitis model [11,12,13,14,15]. Since oxidative stress increases CDK5 activity to activate the NF-κB-mediated pathway [56,57] and CDDO-Me acts as an antioxidant and a NF-κB inhibitor [10], it is plausible that the antioxidant properties of EGCG may have a key role in the attenuation of leukocyte infiltration through NF-κB inhibition. Unlike roscovitine and CDDO-Me, however, EGCG can bind to 67LR and inhibit ERK1/2 activation [16,17,23,51]. Therefore, our findings suggest that the preservation of 67LR functionality may be an additional therapeutic approach against neuroinflammation.

On the other hand, the cortical insults caused by trauma, bleeding and infection result in SE and acquired epilepsy [58,59]. Indeed, post-traumatic injury (TBI) is one of the causes of acquired epilepsy [58]. TBI leads to a subsequent neuronal damage resulting from neuroinflammation, which contributes to synchronized hyperexcitability and robust spontaneous seizures [58]. Furthermore, cobalt-induced neocortical injury leads to focal seizures, which are developed into SE induced by homocysteine (a N-methyl-D-aspartate receptor agonist) administration [59]. Cobalt-induced lesions render the cerebral cortex sensitive to BBB disruption induced by homocysteine [59]. Considering these reports, it is likely that SE-induced leukocyte infiltration into the FPC may affect epileptogenic events without vasogenic edema. Further studies are needed to elucidate the roles of leukocyte infiltration and/or vasogenic edema in the neocortex in epileptogenesis.

In the present study, EGCG effectively attenuated SE-induced NF-κB S276 phosphorylation. Under resting condition, NF-κB is sequestered in the cytoplasm through direct binding with the inhibitor of the κB (IκB) family. IκB kinase (IKK) activation phosphorylates IκB, which leads to IκB degradation, liberates NF-κB from the NF-κB-IκB complex and evokes nuclear NF-κB translocation [60]. Interestingly, N-acetylcysteine (NAC, an antioxidant) abolishes nuclear NF-κB translocation by directly inhibiting NF-κB p65 phosphorylation without affecting IκB degradation [61]. Furthermore, CDDO-Me abrogates NF-κB-mediated signaling pathways by direct inhibition of IKK [62]. Considering that EGCG decreases IκB phosphorylation [63,64,65], it is likely that inhibition of NF-κB canonical pathway by regulating IKK activity may also be relevant to the anti-inflammatory properties of EGCG, which would regulate chemokine and cytokine syntheses. With respect to these previous reports. Furthermore, it is postulated that a general mechanism of antioxidants against inflammation may regulate the early step of NF-κB signaling pathway (inhibitions of IKK activation, IκB phosphorylation, IκB degradation and p65 phosphorylation), although their specific targets would be distinct and unidentified.

## 5. Conclusions

**Figure 8:**
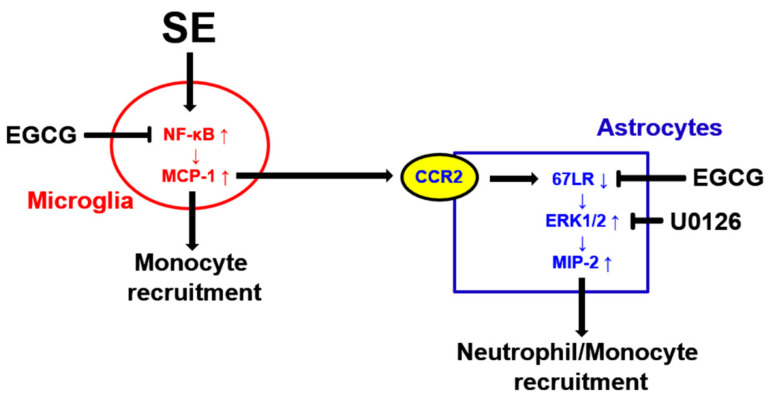
A schematic diagram of the underlying mechanisms of the effect of EGCG on SE-induced leukocyte infiltration. SE led to NF-κB-mediated MCP-1 induction in microglia, which was attenuated by EGCG. Released MCP-1 from microglia recruited blood-derived monocytes and activated MIP-2 upregulation in astrocytes via the CCR2-67LR-ERK1/2 signaling pathway. EGCG and U0126 also attenuated MIP-2 induction in astrocytes by facilitating 67LR-mediated ERK1/2 inactivation, which ameliorated neutrophil/monocyte infiltration.

The present study reveals for the first time that MCP-1 regulated the 67LR-ERK1/2-MIP-2 signaling pathway in astrocytes following SE, which elicited leukocyte infiltration in the FPC independent of vascular permeability. In addition, 67LR neutralization led to leukocyte infiltration via an MCP-1-independent manner under physiological conditions. Furthermore, EGCG attenuated leukocyte infiltration by inhibiting microglial MCP-1 induction and astroglial MIP2 upregulation following SE and 67LR neutralization. Therefore, our findings suggest that the inhibition of MCP-1 induction (in microglia) and/or preservation of 67LR functions (in astrocytes) may be a strategy to mitigate neuroinflammation (Figure 8).

## Figures and Tables

**Table 1:** Primary antibodies used in the present study.

Antigen	Isotype	Hose	Manufacturer (Catalog Number)	Dilution
67LR	IgG	Rabbit	Abcam (ab133645)	1:100
CCR2	IgG	Rabbit	Abcam (ab227015)	1:100
CD68	IgG	Mouse	Abcam (ab31630)	1:100
GFAP	IgG	Mouse	Millipore, Burlington, MA, USA ($MAB3402)	1:4000
IB4	lectin	-	Vector Laboratories, Inc. Newark, CA, USA (B-1205)	1:200
MCP-1	IgG	Mouse	Abcam (ab25124)	1:100
MIP-2	IgG	Rabbit	Invitrogen, Waltham, MA, USA (ARC1074)	1:100
MPO	IgG	Rabbit	Thermo Scientific, Waltham, MA, USA (#RB-373-A)	1:100
NeuN	IgG	Guinea pig	Millipore (#3238431)	1:1000
NF-κB p65	IgG	Rabbit	Abcam (ab16502)	1:2000 (WB *)
p-ERK1/2	IgG	Rabbit	Millipore (#05-767R)	1:100
p-NF-κB p65 S276	IgG	Rabbit	Abcam (ab30623)	1:1000 (WB *)
SMI-71 (endothelial BBB marker)	IgM	Mouse	Covance, Princeton, NJ, USA (#SMI-71R)	1:1000
β-actin	IgG	Mouse	Sigma, St. Louis, MO, USA (#A5316)	1:5000 (WB *)

* WB, Western blot.

## Data Availability

Data is comprised within the article.

## Supplementary Materials

The following supporting information can be downloaded at: https://www.mdpi.com/article/10.3390/antiox12040969/s1, Figure S1: Full-length gel images of Western blot data.
