# Supplementary material for: Epigallocatechin-3-Gallate Attenuates Leukocyte Infiltration in 67-kDa Laminin Receptor-Dependent and -Independent Pathways in the Rat Frontoparietal Cortex following Status Epilepticus
Source: Antioxidants (Basel). 2023 Apr 20;12(4):969. doi: 10.3390/antiox12040969 (PMC10136333; doi:10.3390/antiox12040969)
Supplement: Supplementary file 1 [file antioxidants-12-00969-s001.zip › antioxidants-2257867-supplementary.pdf]

Supplementary Information

Epigallocatechin-3-gallate attenuates leukocyte infiltration in 67-kDa laminin receptor-dependent and -independent pathways in the rat frontoparietal cortex following status epilepticus

Ji-Eun Kim,[1] Duk-Shin Lee,[1] Tae-Cheon Kang[1,*]

[1]Department of Anatomy and Neurobiology, Institute of Epilepsy Research, College of Medicine, Hallym University, Chuncheon 24252, South Korea

* Correspondence to: T. -C. Kang, Department of Anatomy and Neurobiology, College of Medicine, Hallym University, Chuncheon, Kangwon-Do 24252, South Korea; Tel: +82-33-248-2524; Fax: +82-33-248-2525; E-mail: tckang@hallym.ac.kr

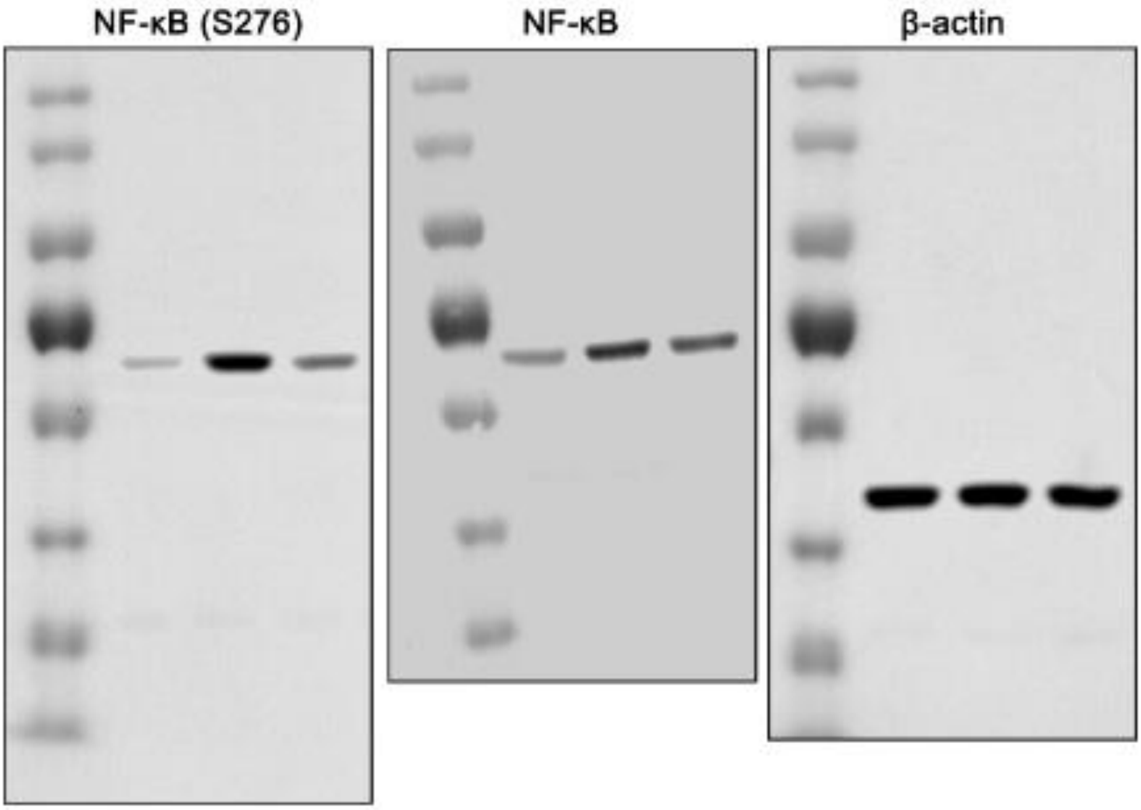

Supplementary Figure S1. Full-length gel images of Western blot data in Fig. 2E.
